# Energy-Efficient Deployment Simulator of UAV-Mounted Base Stations Under Dynamic Weather Conditions

**DOI:** 10.3390/s25123648

**Published:** 2025-06-11

**Authors:** Gyeonghyeon Min, Jaewoo So

**Affiliations:** Department of Electronic Engineering, Sogang University, Seoul 04107, Republic of Korea; ghmin@sogang.ac.kr

**Keywords:** UAV-MBS deployment, geolocation-aware simulator, energy efficiency, hybrid ISA-PSO, weather attenuation

## Abstract

In unmanned aerial vehicle (UAV)-mounted base station (MBS) networks, user equipment (UE) experiences dynamic channel variations because of the mobility of the UAV and the changing weather conditions. In order to overcome the degradation in the quality of service (QoS) of the UE due to channel variations, it is important to appropriately determine the three-dimensional (3D) position and transmission power of the base station (BS) mounted on the UAV. Moreover, it is also important to account for both geographical and meteorological factors when deploying UAV-MBSs because they service ground UE in various regions and atmospheric environments. In this paper, we propose an energy-efficient UAV-MBS deployment scheme in multi-UAV-MBS networks using a hybrid improved simulated annealing–particle swarm optimization (ISA-PSO) algorithm to find the 3D position and transmission power of each UAV-MBS. Moreover, we developed a simulator for deploying UAV-MBSs, which took the dynamic weather conditions into consideration. The proposed scheme for deploying UAV-MBSs demonstrated superior performance, where it achieved faster convergence and higher stability compared with conventional approaches, making it well suited for practical deployment. The developed simulator integrates terrain data based on geolocation and real-time weather information to produce more practical results.

## 1. Introduction

Unmanned aerial vehicle (UAV)-mounted base stations (MBSs) offer a promising way to overcome the limitations of terrestrial BSs due to their high mobility, rapid deployment capabilities, and robust line-of-sight (LoS) channels. The demand for hyper-connectivity and ultra-low latency in fifth-generation (5G) and sixth-generation (6G) technologies makes it difficult for terrestrial base stations (BSs) alone to provide sufficient coverage and flexibility. This issue becomes even more critical in disaster areas, temporary crowding events, rural areas, and places where BSs are often lacking or damaged. In such cases, UAV-mounted BSs stand out as an ideal solution due to their mobility, fast deployment, and flexible positioning [1,2,3].

UAV-MBSs serve as a core infrastructure element in future 6G networks. Their mobility, high operational altitude, and broad coverage allow them to support communication in internet of things (IoT)-concentrated zones, autonomous transportation systems, and smart cities [1]. UAV-MBSs also address key challenges associated with high-frequency bands, such as millimeter-wave or terahertz bands, which will likely be used in beyond-5G and 6G systems. Due to the strong directionality and limited penetration of these bands, UAV-MBSs maintain LoS communication more effectively, ensuring stable links in interreferencenet of flying things (IoFT) environments [2]. Moreover, UAV-MBSs offer a cost-efficient alternative to fixed ground BSs. This is particularly advantageous in rural or sparsely populated areas, where the conventional deployment of BSs is often uneconomical [3].

Some studies have improved the energy efficiency of mobile networks through BS transmission power scaling [4,5]. The authors of reference [4] analyzed the influence of the transmission power and on/off switching of BSs elements on the instantaneous power consumption of BSs. The authors also developed power consumption models for BSs of different generations through on-site measurements performed on real BSs of the second, third, and fourth generations. The authors of reference [5] showed how different BS deployment strategies affect the energy efficiency of the network across five 5G BS installation and operation strategies. The authors demonstrated that the transmission power scaling of the BS can reduce the energy consumption and improve the energy efficiency. From the studies of references [4,5], it is expected that the energy efficiency can be improved by controlling the transmission power of the BS mounted on the UAV in a multi-UAV-MBS network.

Many researchers have shown increasing interest in the deployment and operation of UAV-MBSs across various environments. While some studies addressed single-UAV-MBS scenarios to minimize energy consumption [6,7], others investigated multiple-UAV-MBS scenarios. These include minimizing the number of UAV-MBSs required to provide coverage [8,9,10,11,12,13,14], maximizing the coverage area or the served user equipment (UE) devices [15,16,17,18], and improving the quality of service (QoS) satisfaction [19,20,21]. In addition, some studies aimed to maximize the minimum data rate across all UE [22,23], while others considered multiple objectives simultaneously [24,25].

Given the wide range of problem formulations, researchers have explored various optimization approaches beyond traditional algorithms, such as greedy and clustering-based methods. For instance, the authors of references [6,7] applied variable separation to minimize energy consumption under a given transmission mission for a single UAV-MBS. They determined the optimal transmission power and suboptimal altitude using mathematical analysis and numerical methods, respectively. However, when dealing with multiple UAV-MBSs, the problem becomes more complex. Interactions between multiple variables often lead to non-linear and non-convex optimization problems [22], which are extremely difficult or even impossible to solve analytically.

Hence, many studies on UAV-MBS deployment relied on geometric approaches, simulation-based search methods, or heuristic algorithms. First, using geometric approaches, the authors of reference [9] proposed a specific placement process based on deploying maximal QoS circles to minimize the number of UAV-MBSs. The authors of reference [10] suggested a spiral algorithm that places UAV-MBSs along the boundaries of the UE, starting from the outer region and moving toward the center. In reference [15], the authors determined the positions of UAV-MBSs using the circle-packing theorem to maximize the total coverage area. Second, via simulation-based search approaches, researchers have aimed to maximize the ratio of effective UAV-MBS coverage area using methods such as a random walk algorithm in reference [16] and the simulation of various configurations in reference [17], including the number, location, and transmission power. Third, heuristic algorithms are widely used to solve complex optimization problems by applying experience-based rules or search strategies designed to produce solutions that are sufficiently effective. Instead of ensuring global optimality, these methods focus on practical feasibility and typically yield suboptimal results [26,27]. Based on this approach, the authors of reference [20] reformulated the problem of maximizing UE that satisfy a minimum QoS rate as an NP-hard 0–1 multiple knapsack problem. The authors of references [22,23] applied an iterative algorithm that separates the process into phases to maximize the minimum expected rate between transmitter–receiver pairs by optimizing the UAV-MBS placement.

While heuristic algorithms offer problem-specific strategies, metaheuristic algorithms provide a more general and adaptive framework inspired by natural phenomena. These methods can escape local optima and efficiently explore large search spaces [28]. The authors of reference [18] applied a genetic algorithm (GA) to maximize the UE that meets QoS requirements. The authors of references [19,21] adopted simulated annealing (SA) and particle swarm optimization (PSO), respectively, to maximize the total QoS satisfaction among the UE. In reference [25], the authors introduced a GA-based approach to solve a multi-objective problem that aimed to improve QoS satisfaction while reducing the overall energy consumption. The authors of reference [13] proposed a hybrid method combining GA and PSO to minimize the number of UAV-MBSs, subject to constraints on the coverage ratio and communication quality.

Although many studies have explored suitable algorithms from various perspectives and objectives, only a few have explicitly defined the energy efficiency of the entire network as an objective function and directly optimized it in multi-UAV-MBS environments. Moreover, most of these works do not consider real-world geographical or meteorological conditions in the problem of UAV-MBS placement. In references [29,30], the authors proposed methods based on propagation attenuation modeling under various weather conditions—such as rainfall, snowfall, atmospheric scattering, and fog—to select the maximum operational frequency and to recover coverage, respectively. The study of reference [31] considered both weather-induced attenuation and energy efficiency in its evaluation. However, the authors of references [29,30,31] focused on single-UAV-MBS scenarios, without addressing the optimization of UAV-MBS placement. Because high-frequency communication is highly vulnerable to weather-induced attenuation—especially under extreme conditions, such as tropical heat, humidity, and sudden heavy rainfall (e.g., squalls, downpours)—it is essential to consider both regional features and real-time weather conditions to enable the dense deployment and rapid repositioning of UAV-MBSs [29,30,31,32,33].

From the perspective of simulator development, various graphic user interface (GUI)-based simulators have been developed to enable operators to simulate multi-UAV-MBS network environments. These simulators can be categorized into two types depending on their objectives and functionalities. The first type of simulators is designed to analyze the network performance under varying UAV-MBS placements and trajectories [34,35,36,37]. The second type of simulators focuses on determining the UAV-MBS deployment to meet specific communication performance requirements [38,39,40]. The authors of reference [34] combined UAV-MBS flight simulation with network simulation to represent the interaction between UAV-MBS trajectories and network states. Similarly, the authors of reference [35] developed an integrated simulator to analyze how flight paths influence the network performance. In reference [36], the authors incorporated models for energy consumption, sensors, and peripheral devices, and evaluated how UAV-MBS mobility affects performance metrics, such as throughput and latency. The authors of reference [37] extended the simulator by integrating an intelligent reflecting surface (IRS) module. On the other hand, the authors of reference [38] developed a UAV-MBS deployment simulator with three primary objectives: maximizing the user coverage, minimizing the number of UAV-MBSs, and reducing the flight time required to satisfy user demand. The authors of reference [39] also proposed a similar simulator that incorporates user demand types and behavioral patterns to minimize the coverage gaps, enhance the QoS satisfaction, and improve the energy efficiency. The authors of reference [40] developed a custom simulator framework that determines UAV-MBS BS locations and transmission power levels to maximize the sum-rate while supporting prioritized user access. Although the simulators in references [38,39] incorporate geographic data to reflect terrain characteristics, few existing simulators take both the terrain and weather conditions into account simultaneously in UAV-MBS deployments.

In this paper, we propose an energy-efficient UAV deployment scheme in a multi-UAV-MBS network under dynamic weather conditions. The goal of this study was to extend the battery lifetime of UAVs, where each UAV was powered by an electrical battery that was shared with the mounted BS. By improving the energy efficiency and reducing the transmission power, we can increase the operational lifetime of the battery-powered UAVs. However, it is highly challenging to find a useful solution using a single algorithm because the coordinates and transmission powers of UAV-MBSs differ not only in variable characteristics but also in the scales of their solution spaces. To address this, we combined two multi-variable metaheuristic algorithms: SA for determining the coordinates and PSO for determining the transmission powers. This combination enables an efficient and high-performance solution. We further developed a GUI-based simulator that implements the proposed scheme to facilitate practical application. The main contributions of this study are as follows:

- First, we developed an optimization problem that aimed to maximize the average energy efficiency of the entire multi-UAV-MBS network. Unlike prior studies that focused on the coverage or number of UAV-MBSs, our approach incorporated QoS constraints and weather attenuation to obtain a more practical UAV-MBS deployment strategy.
- Second, in order to efficiently solve the problem of determining the 3D coordinates and transmission powers of UAV-MBSs, we introduced a hybrid improved simulated annealing–particle swarm optimization (hybrid ISA–PSO) algorithm and compared its performance against existing methods. The simulation results show that the proposed algorithm achieved faster convergence and higher stability, and in particular, it demonstrated the efficiency of the novel initialization strategy.
- Third, we developed a geolocation-aware simulator based on the proposed algorithm. The simulator integrates terrain data, real-time weather information, and environmental parameters into the optimization process to derive the approximate solution and provide real-time visualization. Network operators can modify various parameters, compare results, and perform detailed analyses.

The remainder of this paper is organized as follows. Section 2 introduces the system model and formulates the optimization problem. Section 3 presents the proposed hybrid ISA-PSO algorithm. In Section 4, we validate the performance of the algorithm through simulation results. Section 5 describes the implementation of a custom simulator based on the proposed method. Finally, Section 6 concludes the paper.

## 2. System Model and Problem Formulation

### 2.1. System Description

**Figure 1:**
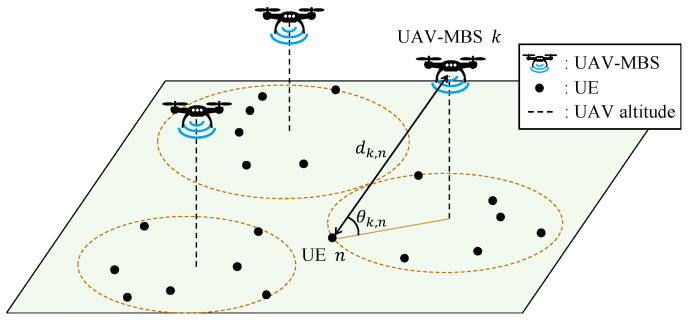
A multi-UAV-MBS network.

We consider a multi-UAV-MBS network serving multiple UE devices, as shown in Figure 1. Here, each UAV is powered by an electrical battery that is shared with the mounted BS. The set of UAV-MBSs is denoted by $K=\left\{1,2,...,K\right\}$ and the set of UE is denoted by $N=\left\{1,2,...,N\right\}$. In the 3D space $S=\{\left(x,y,z\right)\in R^{3}\;|\;x_{\min}\leq x\leq x_{\max},\;y_{\min}\leq y\leq y_{\max},\;z_{\min}\leq z\leq z_{\max}\}$, the coordinate of UAV-MBS *k* is denoted by $c_{k}=\left(x_{k}^{\left(\mathrm{UAV}\right)},y_{k}^{\left(\mathrm{UAV}\right)},z_{k}^{\left(\mathrm{UAV}\right)}\right)\in R^{3}$, ∀k∈K, and the transmission power (in watts) of the BS mounted on the *k*th UAV is represented as $p_{k}$, ∀k∈K. Additionally, we define the set of coordinates and transmission powers as $C=\left(c_{1},c_{2},...,c_{K}\right)$ and $P=\left(p_{1},p_{2},...,p_{K}\right)$, respectively. The coordinate of UE *n* is denoted by $u_{n}=\left(x_{n}^{\left(\mathrm{UE}\right)},y_{n}^{\left(\mathrm{UE}\right)},z_{n}^{\left(\mathrm{UE}\right)}\right)\in R^{3}$, ∀n∈N, and its QoS requirement in terms of the data rate (in bps) is represented as $q_{n}$, ∀n∈N. Each $q_{n}$ belongs to one of |Q| groups, where Q is the set of required data rates.

We define an indicator function, $l_{k,n}$, that represents the connection between UE *n* and UAV-MBS *k* as follows:

(1) $$\begin{array}{c} l_{k,n}=\left\{\begin{array}{cc} 1 & ,\;ifd_{k,n}=\min_{k^{\prime }\in K}\;d_{k^{\prime },n}\\ 0 & ,\;\mathrm{otherwise}, \end{array}\right. \end{array}$$

where $d_{k,n}=∥c_{k}-u_{n}∥$ represents the Euclidean distance between UE *n* and UAV-MBS *k*. Furthermore, we define the set of UE served by UAV-MBS *k* as $L_{k}=\left\{n|\;l_{k,n}=1\right\},\forall k\in K$.

### 2.2. Channel Model

Due to the fact that UAV-MBSs can communicate with UE in various regional environments, a path loss model that accounts for such conditions is required. In most cases, UAV-MBSs operate in the air, and thus, factors such as the altitude, elevation angle, and weather conditions significantly affect the communication quality. Therefore, we adopted a path loss model that combines the air-to-ground model [41] with additional weather attenuation factors [29,31]. Long term evolution (LTE) technology was assumed for the BS because of the path loss model of reference [41]. The path loss between UAV-MBS *k* and UE *n* (in dB) is given by

(2) $$\begin{array}{c} PL\left(d_{k,n}\right)=20\log_{10}\left(\frac{4\pi f_{c}d_{k,n}}{c}\right)+P_{k,n}^{\left(\mathrm{LoS}\right)}\eta_{\mathrm{LoS}}+P_{k,n}^{\left(\mathrm{NLoS}\right)}\eta_{\mathrm{NLoS}}+\frac{\beta^{\left(W\right)}d_{k,n}}{1000}, \end{array}$$

where $f_{c}$ is the carrier frequency in Hz, and *c* is the speed of light in m/s. $P_{k,n}^{\left(\mathrm{LoS}\right)}$ and $P_{k,n}^{\left(\mathrm{NLoS}\right)}$ represent the probabilities of line-of-sight (LoS) and non-line-of-sight (NLoS) communication between UAV-MBS *k* and UE *n*, respectively. $\eta_{\mathrm{LoS}}$ and $\eta_{\mathrm{NLoS}}$ denote the additional path loss in dB under LoS and NLoS conditions. Additionally, $\beta^{\left(W\right)}$ represents the total weather attenuation factor (in dB/km). The $P_{k,n}^{\left(\mathrm{LoS}\right)}$ and $P_{k,n}^{\left(\mathrm{NLoS}\right)}$ can be calculated as follows [41]:

(3) $$\begin{array}{ccc} P_{k,n}^{\left(\mathrm{LoS}\right)} & = & \frac{1}{1+a\exp\left(-b\left(\theta_{k,n}-a\right)\right)}, \end{array}$$

(4) $$\begin{array}{ccc} P_{k,n}^{\left(\mathrm{NLoS}\right)} & = & 1-P_{k,n}^{\left(\mathrm{LoS}\right)}, \end{array}$$

where *a* and *b* are environment-dependent constants and $\theta_{k,n}$ is the elevation angle in degrees between UAV-MBS *k* and UE *n*, which is computed using

(5) $$\begin{array}{c} \theta_{k,n}=\frac{180}{\pi }\tan^{-1}\left(\frac{|z_{k}^{\left(\mathrm{UAV}\right)}-z_{n}^{\left(\mathrm{UE}\right)}|}{\sqrt{{\left(x_{k}^{\left(\mathrm{UAV}\right)}-x_{n}^{\left(\mathrm{UE}\right)}\right)}^{2}+{\left(y_{k}^{\left(\mathrm{UAV}\right)}-y_{n}^{\left(\mathrm{UE}\right)}\right)}^{2}}}\right). \end{array}$$

In this study, we consider weather attenuation caused by rain, snow, and atmospheric refraction, i.e., $\beta^{\left(W\right)}=\beta_{\mathrm{rain}}+\beta_{\mathrm{snow}}+\beta_{\mathrm{atmos}}$, and each component is calculated as follows [32,33,42]:

(6) $$\begin{array}{ccc} \beta_{\mathrm{rain}} & = & k_{r}R_{r}^{\alpha_{r}}, \end{array}$$

(7) $$\begin{array}{ccc} \beta_{\mathrm{snow}} & = & 0.00349\left(\frac{R_{s}^{1.6}}{\lambda_{c}^{4}}\right)+0.00224\left(\frac{R_{s}}{\lambda_{c}}\right), \end{array}$$

(8) $$\begin{array}{ccc} \beta_{\mathrm{atmos}} & = & 0.1820\left(\frac{f_{c}}{10^{9}}\right)\left(N_{\mathrm{Oxygen}}^{\prime \prime }+N_{\mathrm{Watervapour}}^{\prime \prime }\right), \end{array}$$

where $R_{r}$ represents the rainfall rate in mm/h, and $k_{r}$ and $\alpha_{r}$ are frequency-dependent constants specified in reference [32]. Similarly, $R_{s}$ denotes the snowfall rate in mm/h, and $\lambda_{c}=\left(c/f_{c}\right)\times 10^{2}$ represents the wavelength in cm. The terms $N_{\mathrm{Oxygen}}^{\prime \prime }$ and $N_{\mathrm{Watervapour}}^{\prime \prime }$ correspond to the imaginary parts of the frequency-dependent complex refractivity of oxygen and water vapor, respectively, and they can be computed from [33]. In particular, we used the water vapor pressure *e* in hPa from the temperature and relative humidity in the computation of $N_{\mathrm{Oxygen}}^{\prime \prime }$ and $N_{\mathrm{Watervapour}}^{\prime \prime }$ as follows [43]:

(9) $$\begin{array}{c} e=\frac{\rho (273.15+t)}{216.7}=\left(\frac{\mathrm{RH}}{100}\right)\cdot A\cdot \exp\left(\frac{mt}{T_{n}+t}\right), \end{array}$$

where ρ is the water vapor density in $g/m^{3}$, *t* is the temperature in °C, and RH is the relative humidity in %. The constants are given by A=6.112 hPa, m=17.62, and $T_{n}=243.12$ °C. Weather-induced attenuation effects are more pronounced in high-frequency bands, such as millimeter wave and terahertz. Nevertheless, incorporating these effects improves the accuracy of the path loss estimation. Therefore, in Section 4, we evaluate the algorithm using only the A2G model. Then, in Section 5, we integrate both the A2G model and the weather attenuation model to obtain more realistic and practical results.

### 2.3. Problem Formulation

In a multi-UAV-MBS network, UAV-MBSs share the same carrier frequency $f_{c}$. Each UAV-MBS independently operates with its transmission power; let $p_{k}$ denote the transmission power of UAV-MBS *k*. Hence, the UE experiences interference from other UAV-MBSs. The received signal-to-interference-plus-noise ratio (SINR) $\gamma (d_{k,n})$ at UE *n* from UAV-MBS *k* is given by

(10) $$\begin{array}{c} \gamma \left(d_{k,n}\right)=\frac{p_{k}\cdot 10^{-PL(d_{k,n})/10}}{\sum_{k^{\prime }=1,\;k^{\prime }\neq k}^{K}\;p_{k^{\prime }}\cdot 10^{-PL(d_{k^{\prime },n})/10}\;+\sigma_{N}^{2}}, \end{array}$$

where $\sigma_{N}^{2}=N_{0}B$ represents the noise power, $N_{0}$ is the noise power spectral density, and *B* is the channel bandwidth. Our objective was to maximize the average energy efficiency $\bar{\eta }$ of the entire network while satisfying each UE device’s data rate requirement. The optimization problem that determines the 3D placements and transmission powers of UAV-MBSs can then be expressed as follows:

(11) $$\begin{array}{c} \left(C^{*},\;P^{*}\right)=\underset{C,\;P}{\mathrm{argmax}}\;\bar{\eta }=\frac{1}{K}\sum_{k=1}^{K}\frac{\frac{1}{|L_{k}|}\sum_{n\in L_{k}}B\log_{2}\left(1+\gamma (d_{k,n})\right)}{p_{k}}, \end{array}$$

s.t.

(12a) $$\begin{array}{cc} \; & c_{k}\in S\;,\;\forall k\in K, \end{array}$$

(12b) $$\begin{array}{cc} \; & p_{\min}\leq p_{k}\leq p_{\max}\;,\;\forall k\in K, \end{array}$$

(12c) $$\begin{array}{cc} \; & \gamma \left(d_{k,n}\right)\geq \gamma_{\mathrm{th},n}\;,\;\forall k\in K,\;\forall n\in L_{k}, \end{array}$$

(12d) $$\begin{array}{cc} \; & \sum_{k=1}^{K}\sum_{n=1}^{N}l_{k,n}=N, \end{array}$$

where $C=\left(c_{1},c_{2},...,c_{K}\right)$ and $P=\left(p_{1},p_{2},...,p_{K}\right)$ denote the set of the coordinates and transmission powers of the UAV-MBSs, respectively. $\gamma_{\mathrm{th},n}=10\log_{10}\left(2^{q_{n}/B}-1\right)$ is the threshold SINR of UE *n* and $d_{k,n}=∥c_{k}-u_{n}∥$ is the Euclidean distance between UE *n* and UAV-MBS *k*. Constraint (12a) ensures that each UAV-MBS operates within the designated space, while constraint (12b) guarantees that the transmission power of each BS mounted on the UAV remains within the allowable range. Additionally, constraint (12c) ensures that each UE meets its QoS requirement, and constraint (12d) ensures that each UE connects to only one UAV-MBS.

## 3. Proposed Algorithm

### 3.1. Overview of the Proposed Hybrid ISA-PSO

From (11), determining the optimal 3D placements and transmission powers of UAV-MBSs is a highly complex task. Due to the interdependence between multiple variables, the problem is both non-linear and non-convex [20,22]. As a result, finding a global optimal solution theoretically is intractable in practice. To tackle this challenge, we employed a metaheuristic algorithm, which does not guarantee global optimality nor quantifiable closeness to the optimal solution. Nevertheless, the metaheuristic algorithms are known to be effective for exploring high-dimensional and complex search spaces, particularly in solving optimization problems with multiple variables [18,21]. Moreover, it enables the discovery of feasible and efficient configurations that satisfy the given constraints and demonstrate good performance empirically.

**Figure 2:**
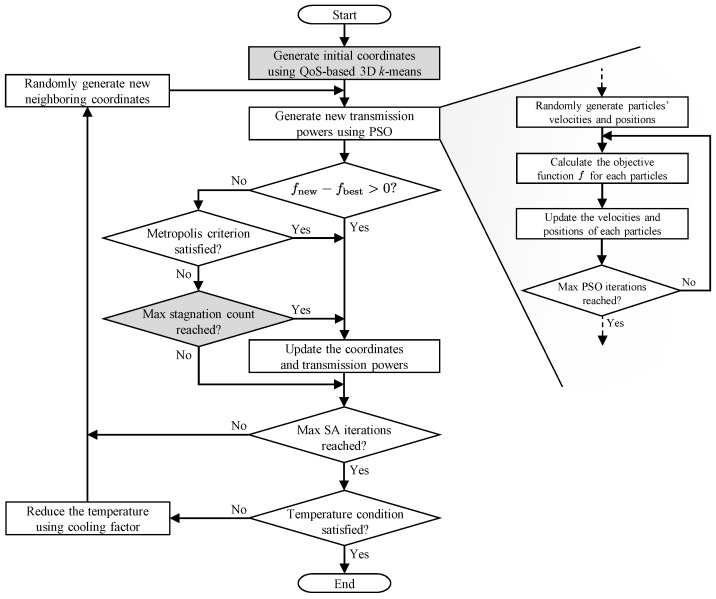
Flowchart of the proposed hybrid ISA-PSO algorithm.

We adopted a hybrid approach that applies SA to determine the coordinates of UAV-MBSs and PSO to determine their transmission powers. The PSO is inspired by swarm intelligence in nature, where each particle explores the solution space by reflecting both its own experience and that of its neighbors [21,44]. The SA, based on a statistical physics model, performs probabilistic exploration at a high initial temperature and gradually converges as the system cools [45]. Due to these characteristics, the PSO is well suited for continuous optimization problems and the SA is effective at escaping local optima. However, the SA is highly sensitive to the quality of the initial solution, which is a critical limitation [45]. To address this, we introduce a new initialization step, named QoS-based 3D k-means initialization, which is based on the QoS levels of the UE. In addition, we used a stagnation count mechanism to further enhance the SA’s ability to escape local optima. The overall flow of the proposed algorithm is illustrated in Figure 2. Detailed descriptions of the main blocks of the flowchart are given in Section 3.2 and Section 3.3.

### 3.2. QoS-Based 3D *k*-Means Initialization

In Figure 2, the box of QoS-based 3D k-means initialization determines the initial positions of UAV-MBSs based on the distribution of UE and its QoS requirements. First, we constructed a coordinate system, where each UE device’s two-dimensional (2D) location is used as the *x*- and *y*-coordinates, and its QoS requirement is assigned as the *z*-coordinate, forming the dataset $(x_{n}^{\left(\mathrm{UE}\right)},y_{n}^{\left(\mathrm{UE}\right)},q_{n}),\forall n\in N$. As the value ranges of different dimensions vary significantly, we normalized the data to ensure balanced clustering using the following transformation:

(13) $$\begin{array}{c} {\hat{x}}_{n}^{\left(\mathrm{UE}\right)}=\frac{x_{n}^{\left(\mathrm{UE}\right)}-\mu_{x}}{\sigma_{x}},\;{\hat{y}}_{n}^{\left(\mathrm{UE}\right)}=\frac{y_{n}^{\left(\mathrm{UE}\right)}-\mu_{y}}{\sigma_{y}},\;{\hat{q}}_{n}=\frac{q_{n}-\mu_{q}}{\sigma_{q}}, \end{array}$$

where $\mu_{x},\;\mu_{y}$, and $\mu_{q}$ represent the mean values of the *x*-coordinates, *y*-coordinates, and QoS requirements of the UE, respectively. Similarly, $\sigma_{x},\;\sigma_{y}$, and $\sigma_{q}$ denote their corresponding standard deviations. Using these, we normalized the data so that each dimension had a mean of 0 and a standard deviation of 1, which resulted in the transformed coordinates ${\hat{u}}_{n}=\left({\hat{x}}_{n}^{\left(\mathrm{UE}\right)},{\hat{y}}_{n}^{\left(\mathrm{UE}\right)},{\hat{q}}_{n}\right),\forall n\in N$. This normalization ensured that all dimensions contributed equally to the clustering process and prevented any single feature from dominating due to scale differences.

Second, we performed k-means clustering to divide the UE into *K* clusters. The process consisted of the following steps:

- Step 1: Randomly select *K* initial cluster centroids, ${\hat{c}}_{k}^{\left(0\right)}=\left({\hat{x}}_{k}^{\left(\mathrm{UAV}\right)},{\hat{y}}_{k}^{\left(\mathrm{UAV}\right)},{\hat{q}}_{k}\right),\;\forall k\in K$.
- Step 2: Each coordinate ${\hat{u}}_{n}$ is assigned to the closest cluster centroid based on the Euclidean distance, i.e., cluster *k* set $S_{k}^{\left(t\right)}=\{{\hat{u}}_{n}|\;{\mathrm{argmin}}_{k^{\prime }\in K}\left|\right|{\hat{u}}_{n}-{\hat{c}}_{k^{\prime }}^{\left(t-1\right)}\left||\right\}$.
- Step 3: Compute the new centroid for each cluster by averaging the coordinates of the UE devices assigned to it, i.e., ${\hat{c}}_{k}^{\left(t\right)}=\frac{1}{|S_{k}^{\left(t\right)}|}\sum_{{\hat{u}}_{n}\in S_{k}^{\left(t\right)}}{\hat{u}}_{n},\;\forall k\in K$.
- Step 4: Repeat until the the total change in the centroid positions falls below a threshold (in this study, we set this threshold to $10^{-4}$), i.e., $\sum_{k=1}^{K}\left|\right|{\hat{c}}_{k}^{\left(t\right)}-{\hat{c}}_{k}^{\left(t-1\right)}\left|\right|<10^{-4}$.

After completing the clustering process, we converted the normalized coordinates back to the original coordinate system using the following denormalization process:

(14) $$\begin{array}{c} x_{k}^{\left(\mathrm{UAV}\right)}=\sigma_{x}{\hat{x}}_{k}^{\left(\mathrm{UAV}\right)}+\mu_{x},\;y_{k}^{\left(\mathrm{UAV}\right)}=\sigma_{y}{\hat{y}}_{k}^{\left(\mathrm{UAV}\right)}+\mu_{y},\;q_{k}=\sigma_{q}{\hat{q}}_{k}+\mu_{q}. \end{array}$$

Finally, we assigned lower altitudes for UAV-MBSs near the UE devices with higher QoS requirements and higher altitudes for UAV-MBSs near the UE devices with lower QoS requirements. The initial $z_{k}^{\left(\mathrm{UAV}\right)}$ for each UAV-MBS is determined as follows:

(15) $$\begin{array}{c} z_{k}^{\left(\mathrm{UAV}\right)}=z_{\max}-\frac{q_{k}-\min\left(Q\right)}{\max(Q)-\min(Q)}\times \left(z_{\max}-z_{\min}\right). \end{array}$$

The initial positioning process corresponds to lines 1–4 of Algorithm 1. Line 1 of Algorithm 1 constructs the initial coordinates of UEs, where the 3D coordinates consist of the x- and y- coordinates of each UE and the QoS requirement as the z-coordinate. Line 2 determines the *K* cluster centers and then denormalizes these center points. Lines 3 and 4 convert the data back to the 3D location coordinate system.
**Algorithm 1** Initial Solution of Hybrid ISA-PSO Algorithm**Input:** UE positions ${\left\{u_{n}\right\}}_{\forall n\in N}$ and QoS requirements ${\left\{q_{n}\right\}}_{\forall n\in N}$**Output:** UAV-MBS initial coordinates $C_{\mathrm{ini}}$, transmission powers $P_{\mathrm{ini}}$, objective function value $f_{\mathrm{ini}}$**Set parameters:** Initial and final inertia coefficients $w_{i},w_{f}$; cognitive and special coefficients  $c_{1},c_{2}$; number of particles *M*; maximum PSO iteration $I_{\max}^{\left(\mathrm{PSO}\right)}$ 1:Replace *z*-coordinate of each $u_{n}$ with $q_{n}$ and normalize it to obtain ${\left\{{\hat{u}}_{n}\right\}}_{\forall n\in N}$ using (13) 2:Determine cluster centers ${\left\{{\hat{c}}_{k}\right\}}_{\forall k\in K}$ by performing the *QoS-based 3D k-means initialization* with *K* clusters on ${\left\{{\hat{u}}_{n}\right\}}_{\forall n\in N}$, then denormalize it to obtain ${\left\{c_{k}\right\}}_{\forall k\in K}$ using (14) 3:Revert *z*-coordinate of $c_{k}$ to $z_{k}$, ∀k∈K, using (15) 4:Set $C_{\mathrm{ini}}=\left(c_{1},c_{2},...,c_{K}\right)$ 5:Generate random transmission power particles ${\left\{x_{m}\right\}}_{m=1}^{M}$ and velocities ${\left\{v_{m}\right\}}_{m=1}^{M}$ 6:Initialize ${\left\{{\mathrm{pbest}}_{m}\right\}}_{m=1}^{M}\leftarrow {\left\{x_{m}\right\}}_{m=1}^{M}$ and $\mathrm{gbest}\leftarrow \underset{{\mathrm{pbest}}_{m}}{\mathrm{argmax}}\;f\left(C_{\mathrm{ini}},{\mathrm{pbest}}_{m}\right)$ 7:**for** i=1 to $I_{\max}^{\left(\mathrm{PSO}\right)}$ **do** 8:    Calculate the objective function ($f=\bar{\eta }+\phi_{p}$) for each particle 9:    Update ${\left\{v_{m}\right\}}_{m=1}^{M}$ and ${\left\{x_{m}\right\}}_{m=1}^{M}$, sequentially using (18) and (19)  10:    ${\mathrm{pbest}}_{m}\leftarrow x_{m},\;\forall m\;\;\mathrm{such}\;\mathrm{that}\;\;f\left(C_{\mathrm{ini}},x_{m}\right)>f\left(C_{\mathrm{ini}},{\mathrm{pbest}}_{m}\right)$  11:    $\mathrm{gbest}\leftarrow \underset{{\mathrm{pbest}}_{m}}{\mathrm{argmax}}\;f\left(C_{\mathrm{ini}},{\mathrm{pbest}}_{m}\right)$  12:**end for**  13:$P_{\mathrm{ini}}\leftarrow \mathrm{gbest},f_{\mathrm{ini}}\leftarrow f\left(C_{\mathrm{ini}},\mathrm{gbest}\right)$

### 3.3. Hybrid ISA-PSO Algorithm for Iterative Searching

#### 3.3.1. Modified Objective Function

The proposed algorithm iteratively searches for an approximate solution by alternating between the PSO and SA phases. The PSO phase determines the transmission powers of the BSs mounted each UAV for a given set of UAV-MBSs coordinates, and the SA phase adjusts the positions of the UAV-MBSs. By repeating this process, the algorithm gradually improves the solution.

To apply this metaheuristic algorithm, instead of solving (11) directly, we reformulated the problem by incorporating constraints (12c) and (12d) as the penalty functions. The modified objective function of (11) can be expressed as follows:

(16) $$\left(C^{*},\;P^{*}\right)=\underset{C,\;P}{\mathrm{argmax}}\;\left(\bar{\eta }+\phi_{p}\right),$$

subject to constraints (12a) and (12b), where $\phi_{p}$ is the penalty function, which is defined as follows:

(17) $$\phi_{p}=\left\{\begin{array}{cc} -\infty & ,\exists k\in K,n\in L_{k}\;s.t.\;\gamma \left(d_{k,n}\right)<\gamma_{\mathrm{th},n}\\ \; & \;\mathrm{or}\;\sum_{k=1}^{K}\sum_{n=1}^{N}l_{k,n}>N\\ 0 & ,\mathrm{otherwise}. \end{array}\right.$$

#### 3.3.2. PSO Phase

To determine the transmission power of each BS mounted on the UAV for a given set of coordinates C, the PSO algorithm stores possible transmission power candidate values in the form of vectors and iteratively updates these vectors to find a candidate with a higher energy efficiency. Here, we refer to each transmission power vector as a *particle*. That is, the term *particle* is not related to any UE cluster or group. Each particle *m* is represented as $x_{m}=\left(p_{1}^{\left(m\right)},p_{2}^{\left(m\right)},\ldots ,p_{K}^{\left(m\right)}\right),\forall m\in \left\{1,2,\ldots ,M\right\}$, where *M* denotes the total number of particles, and $p_{k}^{\left(m\right)}$ represents the transmission power of UAV-MBS *k* in particle *m*. In addition, the transmission powers of each UAV-MBS within a particle are updated individually. The update is represented by the velocity vector $v_{m}=\left(v_{1}^{\left(m\right)},v_{2}^{\left(m\right)},\ldots ,v_{K}^{\left(m\right)}\right)$, where $v_{k}^{\left(m\right)}$ denotes the update corresponding to the $p_{k}^{\left(m\right)}$. To avoid ambiguity, in the remainder of this paper, we refer to $x_{m}$ and $v_{m}$ as particle *m* and its velocity, respectively. Each particle $x_{m}\in R^{K}$ and velocity $v_{m}\in R^{K}$ are randomly generated within the ranges of $\left[p_{\min},p_{\max}\right]$ and $\left[-v_{\max},v_{\max}\right]$, respectively, where $v_{\max}=0.1\left(p_{\max}-p_{\min}\right)$. Each particle evaluates the modified objective function $f=\left(\bar{\eta }+\phi_{p}\right)$ and updates its personal best, ${\mathrm{pbest}}_{m}\in R^{K}$, and the global best, $\mathrm{gbest}\in R^{K}$. Then, each particle updates its velocity, as follows [21,44]:

(18) $$\begin{array}{cc} v_{m}\left(t+1\right)= & \left\{w_{i}-\left(w_{i}-w_{f}\right)\left(t/t_{\max}\right)\right\}v_{m}\left(t\right)\\ & +c_{1}r_{1}\left({\mathrm{pbest}}_{m}-x_{m}\left(t\right)\right)+c_{2}r_{2}\left(\mathrm{gbest}-x_{m}\left(t\right)\right), \end{array}$$

(19) $$\begin{array}{cc} x_{m}\left(t+1\right)\; & =x_{m}\left(t\right)+v_{m}\left(t+1\right), \end{array}$$

where $w_{i}$ and $w_{f}$ are the initial and final inertia coefficients, respectively, and $c_{1}$ and $c_{2}$ are the cognitive and special coefficients, respectively. The parameters $r_{1}$ and $r_{2}$ are random weights, $r_{1},r_{2}\in \left[0,1\right]$. In addition, constraints are applied to ensure (12b), which are $v_{m}\left(t+1\right)=\max\left(-v_{\max},\;\min\left(v_{m}\left(t+1\right),v_{\max}\right)\right)$ and $x_{m}\left(t+1\right)=\max\left(p_{\min},\;\min\left(x_{m}\left(t+1\right),p_{\max}\right)\right)$. This iterative process continues for $I_{\max}^{\left(\mathrm{PSO}\right)}$ cycles, during which gbest is continuously updated. The final gbest is then selected as the new P. The initialization of the overall hybrid ISA-PSO algorithm, which determines the initial solution, are detailed in Algorithm 1. Lines 5–12 of Algorithm 1 describe the PSO phase, where the initial values of $x_{m}$ and $v_{m}$ are generated and iteratively updated. Line 13 assigns the final P and the corresponding objective function value after the iteration concludes.

#### 3.3.3. SA Phase

In each iteration, the SA algorithm generates a new set of coordinates $C_{\mathrm{new}}=\left(c_{\mathrm{new},1},c_{\mathrm{new},2},...,c_{\mathrm{new},K}\right)$, where $c_{\mathrm{new},k}=\left(x_{\mathrm{new},k}^{\left(\mathrm{UAV}\right)},y_{\mathrm{new},k}^{\left(\mathrm{UAV}\right)},z_{\mathrm{new},k}^{\left(\mathrm{UAV}\right)}\right),\;\forall k\in K$. The new coordinates are generated by applying a random perturbation to the previous coordinate:

(20) $$x_{\mathrm{new},k}^{\left(\mathrm{UAV}\right)}=x_{k}^{\left(\mathrm{UAV}\right)}+\Delta x_{k},\;y_{\mathrm{new},k}^{\left(\mathrm{UAV}\right)}=y_{k}^{\left(\mathrm{UAV}\right)}+\Delta y_{k},\;z_{\mathrm{new},k}^{\left(\mathrm{UAV}\right)}=z_{k}^{\left(\mathrm{UAV}\right)}+\Delta z_{k},$$

where $\Delta x_{k},\;\Delta y_{k}$, and $\Delta z_{k}$ are randomly sampled within a step size range $\left[-\lambda ,\lambda \right]$. The step size is applied separately for the horizontal ($\lambda^{\left(\mathrm{xy}\right)}$) and vertical ($\lambda^{\left(z\right)}$) directions. Furthermore, we applied the following boundary constraints to ensure (12a), which are $x_{\mathrm{new},k}^{\left(\mathrm{UAV}\right)}=\max\left(x_{\min},\;\min\left(x_{\mathrm{new},k}^{\left(\mathrm{UAV}\right)},x_{\max}\right)\right),\;y_{\mathrm{new},k}^{\left(\mathrm{UAV}\right)}=\max\left(y_{\min},\;\min\left(y_{\mathrm{new},k}^{\left(\mathrm{UAV}\right)},y_{\max}\right)\right)$, and $z_{\mathrm{new},k}^{\left(\mathrm{UAV}\right)}=\max\left(z_{\min},\;\min\left(z_{\mathrm{new},k}^{\left(\mathrm{UAV}\right)},z_{\max}\right)\right)$.

The new solution is evaluated in the PSO phase and its acceptance follows the Metropolis rule. The probability of acceptance, $P_{r}\left[\Delta f\right]$, is compared with a randomly generated criterion r∈[0,1]. The acceptance probability [19] is calculated via

(21) $$P_{r}\left[\Delta f\right]=\exp\left(\frac{\Delta f}{T}\right),$$

where $\Delta f=f_{\mathrm{new}}-f_{\mathrm{best}}$ is the difference of objective function values and *T* is the current temperature. If Δf>0 or $P_{r}\left[\Delta f\right]>r$, then the new solution is accepted, and the temperature is reduced by multiplying it with the cooling rate α. In the SA algorithm, the initial temperature is set as the initial objective function value itself. This eliminates the need for predefined temperature settings, making the process more stable.

The process repeats up to a maximum of $I_{\max}^{\left(\mathrm{SA}\right)}$ iterations at the same temperature. The SA algorithm ends when the temperature falls below a termination threshold, defined as a fraction ϵ of the initial temperature, i.e., $\epsilon T_{\mathrm{ini}}$. To further improve the exploration, we introduced a maximum stagnation count $\zeta_{\max}$. If the best solution remains unchanged for $\zeta_{\max}$ consecutive iterations, the SA algorithm temporarily accepts a non-optimal solution to escape the local optima and explore a broader search space. During the SA phase, the step size λ is dynamically adjusted using an adjustment factor δ, which is $\lambda \leftarrow \min\left(\delta \lambda ,\lambda_{\max}\right)$, ensuring a controlled expansion. This prevents excessive exploration while still allowing a better solution to emerge. The main iteration process of the proposed hybrid ISA-PSO algorithm is outlined in Algorithm 2. Line 1 of Algorithm 2 initializes the variables C, P, and the objective function value using the initial values, and also sets the *T* and ζ for the SA algorithm. Lines 2–4 of Algorithm 2 represent the generation process of neighboring coordinates. Lines 5–13 correspond to the PSO phase for determining the transmission power, which follows the same structure as in Algorithm 1. Lines 14–26 of Algorithm 2 describe the update procedure based on the Δf and $P_{r}\left[\Delta f\right]$. Lines 27–28 of Algorithm 2 implement the temperature reduction using α, and line 29 assigns the final values C and P after the end of the iteration.

### 3.4. Complexity Analysis

The complexity of the proposed algorithm can be expressed as the sum of the complexity of the initialization process of the UAV-MBS positions and the iterative processes of the SA and PSO phases. First, the complexity, $C_{1}$, of the QoS-based 3D k-means initialization can be approximated as follows [46]:

(22) $$\begin{array}{ccc} C_{1} & = & O(N+NKI^{\left(k\right)}+K+K)\\ & \approx & O(NKI^{\left(k\right)}), \end{array}$$

where *N* is the number of UE devices, *K* is the number of UAV-MBSs, and $I^{\left(k\right)}$ is the number of k-means iterations until convergence to a threshold of $10^{-4}$. In (22), the double summation of *K* arises because the cluster center denormalization and the UAV-MBS altitude assignment are respectively executed *K* times. Second, the complexity of the iterative processes of the SA and PSO phases is the product of the number of iterations, the complexity of the PSO phase, and the complexity of the SA phase. The complexity of the PSO phase is proportional to the number of PSO iterations, the number of particles, and the complexity of the objective function. Hence, the complexity of the PSO phase can be expressed as $O(MNKI_{\max}^{\left(\mathrm{PSO}\right)})$ [47], where *M* is the number of particles and $I_{\max}^{\left(\mathrm{PSO}\right)}$ is the number of PSO iterations. The complexity of the SA phase is proportional to the cost of evaluating each neighboring solution and the number of SA iterations. The termination condition for the SA is defined as the temperature falling below a fraction ϵ of the initial temperature under a geometric cooling schedule T←αT. Hence, the number of total SA iterations becomes $\left\lceil \log\epsilon /\log\alpha \right\rceil \times I_{\max}^{\left(\mathrm{SA}\right)}$, where ϵ is the termination threshold factor and $I_{\max}^{\left(\mathrm{SA}\right)}$ is the number of iterations at the same temperature. The complexity of the iterative processes of the SA and PSO phases can then be expressed as follows:

(23) $$\begin{array}{c} C_{2}=O\left(\left\lceil \log\epsilon /\log\alpha \right\rceil I_{\max}^{\left(\mathrm{SA}\right)}\cdot MNKI_{\max}^{\left(\mathrm{PSO}\right)}\right). \end{array}$$

For $I^{\left(k\right)}\ll I_{\max}^{\left(\mathrm{SA}\right)}I_{\max}^{\left(\mathrm{PSO}\right)}$, the total complexity of the proposed algorithm can then be expressed as

(24) $$\begin{array}{ccc} C_{tot} & = & C_{1}+C_{2}\\ & \approx & O\left(\left\lceil \log\epsilon /\log\alpha \right\rceil \cdot MNK\cdot I_{\max}^{\left(\mathrm{SA}\right)}I_{\max}^{\left(\mathrm{PSO}\right)}\right). \end{array}$$

**Algorithm 2** Main Iteration of Hybrid ISA-PSO Algorithm**Input:** UAV-MBS initial coordinates $C_{\mathrm{ini}}$, transmission powers $P_{\mathrm{ini}}$, objective function  value $f_{\mathrm{ini}}$**Output:** UAV-MBS final coordinates $C^{*}$ and transmission powers $P^{*}$**Set parameters:** Cooling rate α; adjustment factor δ; termination threshold factor ϵ;   basic and maximum step sizes $\lambda ,\lambda_{\max}$; maximum stagnation count $\zeta_{\max}$; maximum   SA-iteration $I_{\max}^{\left(\mathrm{SA}\right)}$; initial and final inertia coefficients $w_{i},w_{f}$; cognitive and special   coefficients $c_{1},c_{2}$; number of particles *M*; maximum PSO-iteration $I_{\max}^{\left(\mathrm{PSO}\right)}$ 1:$C_{\mathrm{best}}\leftarrow C_{\mathrm{ini}},\;P_{\mathrm{best}}\leftarrow P_{\mathrm{ini}},\;f_{\mathrm{best}}\leftarrow f_{\mathrm{ini}},\;T_{\mathrm{ini}}\leftarrow f_{\mathrm{ini}},\;T\leftarrow T_{\mathrm{ini}},\;\zeta \leftarrow 1$ 2:**while** $T>\epsilon T_{\mathrm{ini}}$ **do** 3:    **for** i=1 to $I_{\max}^{\left(\mathrm{SA}\right)}$ **do** 4:        Generate random neighboring $C_{\mathrm{new}}$ of previous $C_{\mathrm{best}}$ using (20) 5:        Generate random transmission power particles ${\left\{x_{m}\right\}}_{m=1}^{M}$ and velocities ${\left\{v_{m}\right\}}_{m=1}^{M}$ 6:        Initialize ${\left\{{\mathrm{pbest}}_{m}\right\}}_{m=1}^{M}\leftarrow {\left\{x_{m}\right\}}_{m=1}^{M}$ and $\mathrm{gbest}\leftarrow \underset{{\mathrm{pbest}}_{m}}{\mathrm{argmax}}\;f\left(C_{\mathrm{new}},{\mathrm{pbest}}_{m}\right)$ 7:        **for** j=1 to $I_{\max}^{\left(\mathrm{PSO}\right)}$ **do** 8:           Calculate the objective function ($f=\bar{\eta }+\phi_{p}$) for each particle 9:           Update ${\left\{v_{m}\right\}}_{m=1}^{M}$ and ${\left\{x_{m}\right\}}_{m=1}^{M}$ sequentially using (18) and (19) 10:            ${\mathrm{pbest}}_{m}\leftarrow x_{m},\;\forall m\;\;\mathrm{such}\;\mathrm{that}\;\;f\left(C_{\mathrm{new}},x_{m}\right)>f\left(C_{\mathrm{new}},{\mathrm{pbest}}_{m}\right)$ 11:            $\mathrm{gbest}\leftarrow \underset{{\mathrm{pbest}}_{m}}{\mathrm{argmax}}\;f\left(C_{\mathrm{new}},{\mathrm{pbest}}_{m}\right)$ 12:        **end for** 13:        $P_{\mathrm{new}}\leftarrow \mathrm{gbest},f_{\mathrm{new}}\leftarrow f\left(C_{\mathrm{new}},\mathrm{gbest}\right)$ 14:        Calculate $\Delta f=f_{\mathrm{new}}-f_{\mathrm{best}}$ and acceptance probability $P_{r}\left[\Delta f\right]$ using (21) 15:        **if** Δf>0 **then** 16:            $\left(C_{\mathrm{best}},P_{\mathrm{best}}\right)\leftarrow \left(C_{\mathrm{new}},P_{\mathrm{new}}\right),\;f_{\mathrm{best}}\leftarrow f_{\mathrm{new}},\;\zeta \leftarrow 1$ 17:        **else if** $P_{r}\left[\Delta f\right]>r\in \left[0,1\right]$ **then** 18:            $\left(C_{\mathrm{best}},P_{\mathrm{best}}\right)\leftarrow \left(C_{\mathrm{new}},P_{\mathrm{new}}\right),\;f_{\mathrm{best}}\leftarrow f_{\mathrm{new}},\;\zeta \leftarrow 1$ 19:        **else if** $\zeta >\zeta_{\max}$ **then** 20:            $\lambda \leftarrow \min\left(\delta \lambda ,\lambda_{\max}\right)$ 21:            $\left(C_{\mathrm{best}},P_{\mathrm{best}}\right)\leftarrow \left(C_{\mathrm{new}},P_{\mathrm{new}}\right),\;f_{\mathrm{best}}\leftarrow f_{\mathrm{new}},\;\zeta \leftarrow 1$ 22:        **else** 23:           Keep the solution and objective function value 24:           ζ←ζ+1 25:        **end if** 26:    **end for** 27:    T←αT 28:**end while** 29:$\left(C^{*},P^{*}\right)\leftarrow \left(C_{\mathrm{best}},P_{\mathrm{best}}\right)$

## 4. Simulation Results

We compared the performance of the proposed algorithm with conventional algorithms, such as the combined SA-PSO, SA-only, PSO-only, and random approach algorithms, in terms of the convergence and energy efficiency. Here, for a fair comparison, we assumed normal operating conditions, clear weather, and a typical carrier frequency (2.1 GHz). Because the atmospheric interference and the attenuation are known to be negligible under these conditions [29,32,33], we set the value of $\beta^{\left(W\right)}$ to zero. Although we set $\beta^{\left(W\right)}$ to zero in this simulation, the weather attenuation factor is already incorporated in the path loss model of (2). Moreover, the simulator version 1.0 released in GitHub allows for configuring various weather conditions through a GUI [48]. All UE was assumed to be arbitrarily distributed within a $3000\times 3000\;m^{2}$ flat area of urban environments, setting $z_{n}^{\left(\mathrm{UE}\right)}=0,\forall n\in N$, $\left(a,b\right)=\left(9.61,0.16\right)$, $\left(\eta_{\mathrm{LoS}},\eta_{\mathrm{NLoS}}\right)=\left(1.0,20\right)\;\mathrm{dB}$ [41], and $f_{c}=2.1\;\mathrm{GHz}$. For the SA phase of the proposed algorithm, we set the following: the cooling rate was α=0.8 and the termination threshold factor was $\epsilon =10^{-3}$. In the SA, the geometric cooling schedule was used to reduce the temperature, where the cooling rate α is typically chosen between 0.8 and 0.99 to ensure a gradual reduction in temperature and the termination criterion for the annealing process is often set when the temperature reaches a small fraction (e.g., 0.1%) of its initial value [49]. Moreover, the study of reference [19] showed that setting α=0.8 is effective at solving the UAV-MBS coverage optimization problem. For the PSO phase of the proposed algorithm, we set the following: initial and final inertia coefficients were $\left(w_{i},w_{f}\right)=\left(0.9,0.4\right)$, and the cognitive and special coefficients were $\left(c_{1},c_{2}\right)=\left(2.0,2.0\right)$, which refer to the values used in the studies of references [50,51]. Additionally, we experimentally determined other parameters, such as the step range λ, the adjustment factor δ, and the number of iterations, based on the simulation space range. The simulation parameters are summarized in Table 1, while the algorithm parameters are listed in Table 2.

**Figure 3:**
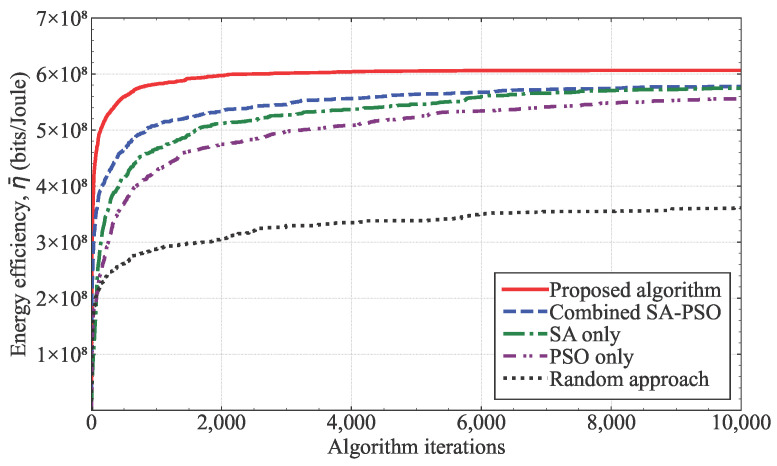
Convergence and energy efficiency of algorithms.

Figure 3 shows the energy efficiency, $\bar{\eta }$, of the algorithms according to the iterations under identical simulation conditions. To ensure a fair comparison between the metaheuristic algorithms, we ran each algorithm 50 times and plotted the average energy efficiency of each algorithm. Our results show that the random-based algorithm demonstrated the worst performance. While the SA algorithm outperformed the PSO algorithm, the combined SA-PSO algorithm demonstrated even more improved performance. The proposed hybrid ISA-SA algorithm significantly outperformed the other algorithms in terms of the convergence speed, stability, and energy efficiency. Compared with the conventional SA-PSO algorithm, which randomly determines the initial positions of UAV-MBSs, the proposed algorithm determines the initial positions of UAV-MBSs based on the distribution of the UE and its QoS requirements. This strategic initialization in the proposed scheme effectively accelerates the convergence. Additionally, the proposed algorithm includes a stagnation count mechanism that enhances the overall performance by helping to escape the local optima. Compared with the conventional SA and PSO algorithms, the proposed algorithm accelerates the convergence by alternately updating the UAV-MBS coordinates and transmission power rather than attempting to optimize all the variables simultaneously.

The computational time of the algorithm was inversely proportional to the convergence speed, and therefore, the proposed algorithm could reduce the computation time due to its fastest convergence speed compared with the conventional algorithms, such as the combined SA-PSO, SA-only, and PSO-only algorithms. The execution time on an actual computer depends on the hardware specifications and the program code optimization. The developed Python-based simulator took several minutes to run on an Intel Core i-7 computer, where the version of the Python is 3.13.2.

**Figure 4:**
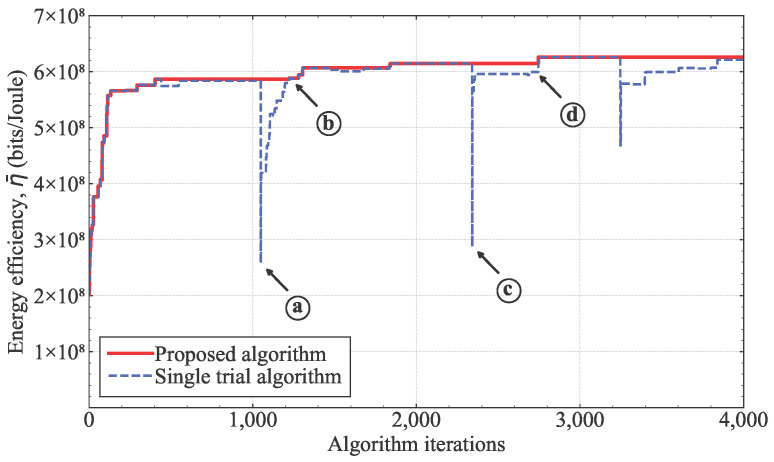
Objective function value for a single trial of the proposed algorithm.

Figure 4 shows that the proposed stagnation count in the hybrid ISA-PSA algorithm was important for escaping the local optima. The blue dashed plot shows the energy efficiency for a single trial of the proposed algorithm, and the red plot shows the final energy efficiency of the proposed algorithm for multiple iterations. In Figure 4, for a single attempt, the algorithm may accept non-optimal solutions (e.g., points ⓐ and ⓒ) depending on the stagnation count, but the algorithm will find a better solution, such as points ⓑ and ⓓ, in subsequent moments. These observations demonstrate that the introduction of the stagnation count helps the algorithm escape local optima and reach improved solutions, thereby enhancing the overall performance of the proposed algorithm.

**Figure 5:**
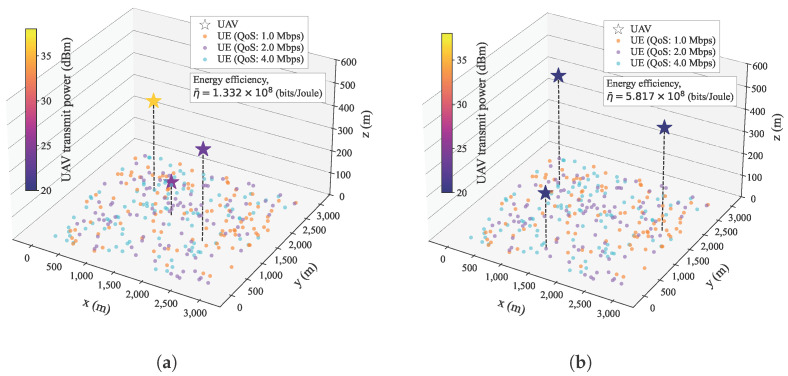
UAV-MBS deployment visualizations in the proposed algorithm. (**a**) Initial UAV-MBS deployment. (**b**) Final UAV-MBS deployment.

Figure 5 shows the effectiveness of the proposed algorithm in solving the energy efficiency maximization problem through the joint determination of UAV-MBS positions and transmission powers. In Figure 5, the circular markers on the x-y plane represent the ground UE, and the colors of the circular markers represent the randomly assigned QoS requirements of the data rates, where the colors orange, purple, and cyan represent the required data rates of 1.0 Mbps, 2.0 Mbps, and 4.0 Mbps, respectively. The star-shaped markers indicate the positions of the UAV-MBSs, and their colors correspond to the transmission powers. Figure 5a presents the initial UAV-MBS deployment for a single trial of the proposed algorithm. As described in Section 3.2, the initial UAV-MBS positions are determined using the 2D coordinates of the centroid derived from the QoS-based 3D clustering, where the UAV-MBS altitudes are set to be inversely proportional to the aggregated QoS requirements. In this simulation configuration, the UAV-MBSs were placed closely together, which resulted in an energy-inefficient deployment. Figure 5b shows the final UAV-MBS deployment after the proposed iterations. Compared with the initial deployment, the UAV-MBSs were more widely distributed and evenly spread across the simulation space. This spatial relocation allowed the UAV-MBSs to provide better services for the UE. As a result, the final deployment achieved a higher energy efficiency.

**Figure 6:**
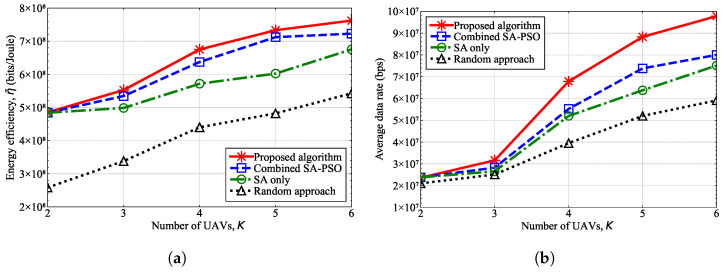
Performance results according to the number of UAV-MBSs when N=100. (**a**) Energy efficiency. (**b**) Average data rate.

Figure 6 shows the performance in terms of the energy efficiency and the average data rate according to the number of UAV-MBSs, *K*, when the number of UE devices was fixed at N=100. As the number of UAV-MBSs increased, the number of UE devices served by a single UAV-MBS decreased, which consequently increased the data rate of the UE and resulted in an increase in the energy efficiency of the UAV-MBS. Additionally, the proposed hybrid ISA-PSO algorithm outperformed other algorithms in terms of all the performance metrics. As previously stated, the random-based algorithm exhibited the worst performance, and although the SA algorithm outperformed the random approach, the combined SA-PSO algorithm showed an even better performance than the SA algorithm.

**Figure 7:**
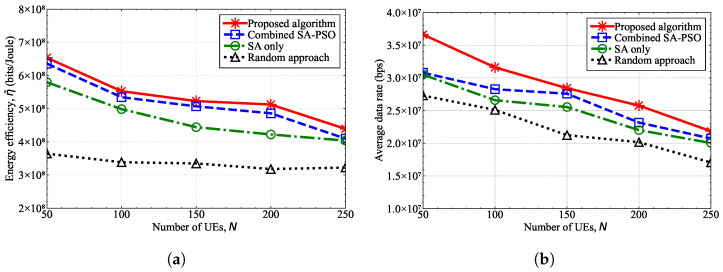
Performance results according to the number of UE devices when K=3. (**a**) Energy efficiency. (**b**) Average data rate.

Figure 7 shows the performance in terms of the energy efficiency and the average data rate according to the number of UE devices, *N*, when the number of UAV-MBSs was fixed at K=3. As the number of UE devices increased in a limited area, each UAV-MBS should serve more UE, thereby causing the data rate of the UE to decrease. Hence, the energy efficiency decreased as the number of UE devices increased. The proposed algorithm outperformed the other algorithms because it consistently found a better solution within the limited number of iterations, which demonstrated that the proposed algorithm is feasible for determining UAV-MBS deployment and the transmission power.

## 5. Simulator Development

We developed a simulator for the deployment of UAV-MBSs in multi-UAV-MBS networks by applying the modified objective function and the proposed hybrid ISA-PSO algorithm. The simulator was implemented using Python 3.10 and used standard libraries, including PyQt6, to build the GUI. The developed simulator is available at the GitHub [48].

The simulator developed in this study provides several additional features compared with conventional UAV-MBS deployment simulators [38,39,40]. The features of conventional simulators are as follows: The simulator introduced in reference [38] reflects the LoS/NLoS path loss based on real geographic information from Google Maps. Similarly, the simulator of reference [39] supports the input of various simulation parameters, such as number of UAV-MBSs and UE devices, UAV-MBS altitude, bandwidth, and traffic types, as well as the input of map data. The simulator of reference [40] determines UAV-MBS locations and transmission power levels to maximize the sum-rate of the network. Unlike existing simulators, the developed simulator utilizes not only the imported map image but also the elevation data of the selected region to assign real height coordinates to the UE. Moreover, the developed simulator takes both the terrain and weather conditions into account simultaneously in UAV-MBS deployments. The main features of the developed simulator are as follows:

- The operator can build arbitrary simulation environments by configuring various parameters, including the number of UAV-MBSs and UE devices, QoS requirements, carrier frequency and bandwidth, geographic location in latitude and longitude, and regional characteristics.
- The simulator performs simulations based on the terrain data and real-time meteorological conditions of the selected area and provides analytical results. Additionally, the operator can control the algorithm’s progress by modifying its hyperparameters.
- The simulator interacts with the operator in real time, i.e., the simulator visualizes UAV-MBS deployments and shows the progress of the algorithm through graphs.

Similar to the simulator developed in this study, the conventional simulators in references [38,39,40] also determine UAV-MBS deployments. However, the performance of the developed simulator was not compared with these conventional simulators due to differences in the objective functions, system model assumptions, and optimization strategies. First, compared with the studies in references [38,39], this study differed in its objective and optimization approach. The objective of our simulator was to determine the 3D positions of UAV-MBSs that maximize the average energy efficiency of the entire UAV-MBS network. The developed simulator employs a hybrid approach, utilizing SA to determine the 3D positions of UAV-MBSs and PSO to optimize the transmission powers of UAV-MBSs. In contrast, the simulator in reference [38] was designed to identify UAV-MBS positions that maximize the number of serviced users, minimize the number of UAV-MBSs required to provide full coverage, or reduce the total flight time for serving users. Furthermore, the simulator in reference [38] adopts a heuristic approach to the optimization problem, where the target area is discretized into a set of UAV-MBS candidate points and the transmission power of each UAV-MBS was assumed to be fixed. The simulator in reference [39], on the other hand, focuses on UAV-MBS trajectories to and from a charging station, aiming to minimize energy consumption, which can be regarded as a path-planning problem. The simulator in reference [39] applies the A* algorithm to solve this UAV-MBS path-planning problem, with the UAV-MBS flight area discretized into grid cells and the energy consumption between adjacent grids formulated as a cost function. The A* algorithm is a commonly used path-planning method and traditionally performs searches on a grid map [52]. Moreover, the simulator in reference [40] was designed to determine UAV-MBS positions that maximize the user association and the weighted sum-rate of users. The simulator in reference [40] employs a Q-learning algorithm, which maximizes a reward function expressed as a sum-rate metric. Here, the Q-learning algorithm is a learning-based strategy, whereas the SA and PSO algorithms used in this study are search-based strategies. Although the performance of the developed simulator was not directly compared with the conventional simulators in references [38,39,40], the effectiveness of the algorithm implemented in the simulator is demonstrated in Figure 3, Figure 6 and Figure 7. The algorithm implemented in the simulator exhibited a significantly faster convergence, as shown in Figure 3, and achieved a higher energy efficiency for the UAV-MBSs, as illustrated in Figure 6 and Figure 7, when compared with the SA-only, PSO-only, and combined SA-PSO algorithms.

**Figure 8:**
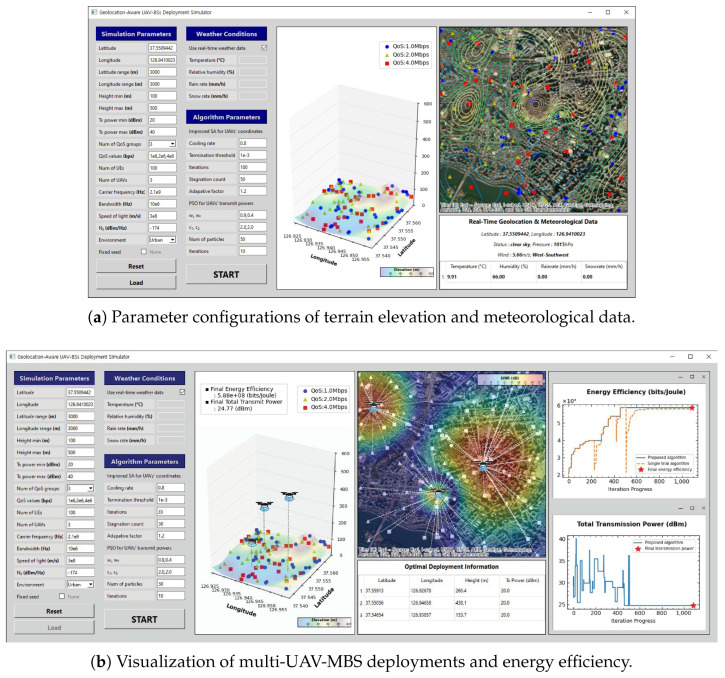
Example of the GUI of the developed simulator.

Figure 8 illustrates the GUI of the developed simulator. Unlike that in Section 4, the simulator computed the path loss by incorporating both the altitudes (*z*-coordinates) of the UE devices and weather attenuation. In Figure 8a, the GUI displays a map of the selected area with contour lines based on the user input and updates the weather conditions. It also provides the 3D distribution of the UE devices, along with their assigned QoS requirements. Figure 8b shows the applied proposed hybrid ISA-PSO algorithm using the specified algorithm parameters to determine the UAV-MBS deployments. It visualizes the resulting SINR map and plots the change in energy efficiency over iterations, enabling the operators to observe the convergence behavior.

The developed simulator excels by integrating real-world data with a flexible environment that allows operators to freely select regions and implement communication settings. This capability is particularly valuable for analyzing millimeter-wave and terahertz communication systems, which are highly sensitive to weather variations. It enables simulations that extend beyond theoretical models by incorporating actual terrain and climate conditions. These features can be effectively utilized, especially in emergency scenarios or in areas of rapid severe weather events. During disaster emergencies, ground BSs often become unavailable, making UAV-MBS coverage a practical alternative. In these cases, the ability to rapidly determine energy-efficient UAV-MBS placements provides a significant advantage. Moreover, in environments prone to abrupt and intense weather—such as tropical squalls or monsoon-driven downpours—climate-induced attenuation can critically degrade the performance. Under these conditions, UAV-MBSs offer a viable solution to complement terrestrial networks. The simulator incorporates these real-world conditions, making it a practical tool with strong potential for real-world deployment beyond theoretical research.

## 6. Conclusions

UAV-mounted BSs are a promising means of providing hyper-connectivity and higher data rates to UE. In particular, given the limited battery life of UAV-MBSs, it is important to increase their energy efficiency in multi-UAV-MBS networks. In this paper, we present a hybrid improved simulated annealing–particle swarm optimization (ISA-PSO) algorithm that determines the 3D placement and transmission power of UAV-MBSs in order to maximize their energy efficiency while satisfying the QoS requirements of UE. Moreover, we included QoS-based 3D k-means initialization to construct the initial positions of the UAV-MBSs in the proposed algorithm, which has been shown to improve the convergence speed. We incorporated the QoS constraints of UE and weather attenuations to achieve a practical UAV-MBS deployment strategy. The proposed hybrid ISA-PSO algorithm significantly outperformed both the SA and PSO algorithms in terms of the convergence speed, the energy efficiency of UAV-MBSs, and the average data rate of UE.

Additionally, we developed a simulator for energy-efficient UAV-MBS deployments. The simulator incorporates geolocation-based terrain and weather data, which improve both the accuracy and practical applicability. User-friendly GUI and real-time visualization enable operators to easily modify various parameters, monitor UAV-MBS deployments, and analyze the performance. The developed simulator demonstrates significant potential for real-world applications, particularly in environments with severe weather conditions or emergency situations.

## Figures and Tables

**Table 1:** Simulation parameters.

Parameter	Value
*x*-range of simulation space, $\left(x_{\min},x_{\max}\right)$	$\left(0,3000\right)\;$ m
*y*-range of simulation space, $\left(y_{\min},y_{\max}\right)$	$\left(0,3000\right)\;$ m
*z*-range of simulation space, $\left(z_{\min},z_{\max}\right)$	$\left(100,500\right)\;$ m
Transmission power-range of UAV-MBSs, $\left(p_{\min},p_{\max}\right)$	$\left(20,40\right)\;\mathrm{dBm}$
Number of UAV-MBSs, *K*	3
Number of UE devices, *N*	100
Set of QoS requirements, Q	$\left\{1M,2M,4M\right\}\;\mathrm{bps}$
Carrier frequency, $f_{c}$	2.1 GHz
Speed of light, *c*	$3\times 10^{8}$ m/s
Noise power spectral density, $N_{0}$	−174 dBm/Hz
Channel bandwidth, *B*	10MHz
Environment-dependent constants, $\left(a,b\right)$	$\left(9.61,0.16\right)$
Additional path loss, $\left(\eta_{\mathrm{LoS}},\eta_{\mathrm{NLoS}}\right)$	$\left(1.0,20\right)\;\mathrm{dB}$

**Table 2:** Algorithm parameters.

Parameter	Value
Cooling rate, α	0.8
Adjustment factor, δ	1.1
Termination threshold factor, ϵ	$10^{-3}$
Horizontal and vertical step range, $\left(\lambda^{\left(\mathrm{xy}\right)},\lambda^{\left(z\right)}\right)$	$\left(100,50\right)$ m
Maximum horizontal and vertical step ranges, $\left(\lambda_{\max}^{\left(\mathrm{xy}\right)},\lambda_{\max}^{\left(z\right)}\right)$	$\left(1500,250\right)$ m
Maximum stagnation count, $\zeta_{\max}$	500
Maximum SA iteration, $I_{\max}^{\left(\mathrm{SA}\right)}$	350
Maximum PSO iteration, $I_{\max}^{\left(\mathrm{PSO}\right)}$	50
Number of particles, *M*	10
Initial and final inertia coefficients, $\left(w_{i},w_{f}\right)$	$\left(0.9,0.4\right)$
Cognitive and special coefficients, $\left(c_{1},c_{2}\right)$	$\left(2.0,2.0\right)$

## Data Availability

Data is contained within the article.
